# Supplementary material for: Chromothripsis during telomere crisis is independent of NHEJ, and consistent with a replicative origin
Source: Genome Res. 2019 May;29(5):737–49. doi: 10.1101/gr.240705.118 (PMC6499312; doi:10.1101/gr.240705.118)
Supplement: Supplemental Material [file supp_gr.240705.118_Supplemental_file_1.zip › contigs/annotated_contigs/DB83/contig.2.DB83_length_708_mean_cov_8.55367231638.docx]

**DB83_length_708_mean_cov_8.55367231638**

```
CAGGGCTGAAGGGGGTAGAGCTATC|AAAAAACCATTCCAAAAATCCGTGAATCCAGGAGCTGGTTTTTTGAAAAGATCAACAAAATTG
                          >chr4:47247040-47247486 + E=1e-253 p=2e-02
ATAGACCGCTAGCAAGACTAATAAAGAAGAAAAGAGAGAAGAATCAAACAGATGCAATAAAAAATGATAAAGGGGATATCACCACTGAT

TCCACAGAAATACAAACTACCATCAGAGAATACTATAAACAGATTCACTTAAGGTTTTTTTCCTGCATCCAATAAGCTAAAATGTAAAA

CACACTATTTCCTAATATAACTTGCAATAAAAAATATCAAGTGACCAATATTAATTTTGTTCAGATAGCCTAACAGTTTTGCTTTGTAA

ATTCTAAGTTCTCAGTTATTCCCTCAGAGAAAGAGTAGGGGATACCAATGTTCAATTATTCATCACGTAAAATAGTCACACAAGGAACT

GAGTTTTCCTTGCTCCCTGGAAAAG|AT|ATATTTATATATATATATAAAATACCACAGTTTCTTTATCCACTTGTTGATTGATGGGAA
                          >chr4:47248392-47248631 + E=5e-129
TTTGGGTTGGTTCCACTATTTTGCAATTGTGAATTGTGCTACTATAAACATGCATATACAAGTATCTTTTTCATATATTGACTTCTTTG

TCTCTGGGTAAATACCCAGTAGTAGAACTACTGGGTCAAATGGTAGTTCTACTTTTAGTTCTTTGAGGAATCTTCACACTGTTTTCCA
```
